# Assessment of indoor radon distribution and seasonal variation within the Kpando Municipality of Volta Region, Ghana

**DOI:** 10.1371/journal.pone.0299072

**Published:** 2024-02-27

**Authors:** Anthony Selorm Kwesi Amable, Francis Otoo, Paul Kingsley Buah-Bassuah, Anthony Kwabena Twum

**Affiliations:** 1 School of Basic and Biomedical Sciences, Department of Basic Sciences, University of Health and Allied Sciences, Ho-Volta, Ghana; 2 School of Physical Sciences, Department of Physics, University of Cape Coast, Cape Coast, Ghana; 3 Radiation Protection Institute, Ghana Atomic Energy Commission, Legon-Accra, Ghana; 4 School of Nuclear and Allied Sciences, University of Ghana, Atomic Campus, Accra, Ghana; Al Mansour University College-Baghdad-Iraq, IRAQ

## Abstract

This study uses CR-39 radon detectors to examine radon distributions, seasonal indoor radon variations, correction factors, and the influence of building materials and characteristics on indoor radon concentration in 120 dwellings. The study also determines the spatial distribution of radon levels using the ArcGIS geostatistical method. Radon detectors were exposed in bedrooms from April to July (R_S_), August to November (D_S_); December to March (H_S_), and January-December (Y_S_) from 2021 to 2022. The result for the radon levels during the weather seasons were; 32.3 to 190.1 Bqm^-3^ (80.9 ± 3.2 Bq/m^3^) for (R_S_), 30.8 to 151.4 Bqm^-3^ (68.5 ± 2.7 Bqm^-3^) for H_S_ and 24.8 to 112.9 Bqm^-3^(61.7 ± 2.1 Bqm^-3^) for D_S_, and 25.2 to 145.2 Bq/m^3^ (69.4 ± 2.7 Bqm^-3^). The arithmetic mean for April to July season was greater than August to November. The correction factors associated with this study ranged from 0.9 to 1.2. The annual effective dose (A_E_) associated with radon data was varied from 0.6 to 4.04 mSv/y (1.8 ± 0.1 mSv/y). The April to July period which was characterized by rains recorded the highest correlation coefficient and indoor radon concentration. Distribution and radon mapping revealed radon that the exposure to the occupant is non-uniformly spread across the studied dwellings. 15.4% of the studied data exceeded WHO reference values of 100 Bq/m^3^. The seasonal variation, dwelling age, and building materials were observed to have a substantial impact on the levels of radon concentration within the buildings.

## Introduction

The inhalation of radon progenies has been studied extensively, revealing that it accounts for over fifty percent of the natural radiation dose received by the general public [1]. Radon is classified as the second main cause of lung cancer after cigarette smoking. It has been characterized as cancerogenic and causes between 3 to 14% of all lung cancer cases reported globally [2–5]. Literature studies and the International Organization on Radiation Protection have reported that exposure to elevated indoor radon levels in enclosed areas such as dwellings can result in a higher risk of lung cancer incidence among the populace [2, 6]. As a result, international organizations have provided recommendations for the protection of people from the radiological effects associated with radon exposure in homes.

Radon concentration levels in dwellings were found to differ from location to location, building to building as a result of the geology, and seasonal factors such as atmospheric pressure, humidity, temperature, building structure, age, and construction materials [7–12]. Several studies have been conducted on the seasonal variability of indoor radon concentration levels [7, 8, 11, 13–15]. Studies conducted globally to determine indoor radon concentration during winter (rainy season) and summer (dry season) [8, 11, 13, 15–17] weather seasons aim to find out which seasonal period contains higher indoor radon levels. The studies conducted in Ghana and abroad reported higher indoor radon levels during the rainy (winter) season which were mainly linked to the windows and doors normally closed due to the rains and little bit colder compared to the dry (summer) season, resulting in an accumulation of radon in the room coming from the soil underneath the building and construction materials [7, 8, 11, 17, 13].

To determine radon distribution, there have been a lot of studies that zone and develop radon maps using radon data and considering other seasonal environmental factors [8, 18]. The purpose of the map is to categorize regions where there is a higher likelihood of elevated radon levels. This map aims to aid in the implementation of preventive measures against radon exposure and also the establishment of resilient building codes. Mapping radon gas will be instrumental in developing tools and strategies to design effective approaches for reducing radon exposure among residents.

There has been a lot of literature on seasonal indoor radon studies, correction factors, and radon mapping [8, 11, 13–15, 18–21]. In Ghana, there have been a lot of studies that reported indoor radon levels [8, 9, 11, 22–26]. Most of the reported indoor radon studies were conducted over relatively short periods [9, 22–26] with few studies having seasonal radon data [8, 11, 13]. Previous radon studies conducted in the study region have primarily focused on indoor radon levels in some dwellings, and there are no known studies that have determined radon levels in dwellings within the study location itself. Furthermore, indoor radon seasonal variation studies conducted in Ghana have typically focused on the rainy and dry weather seasons but have not taken into account the harmattan season. The harmattan season is characterized by the dry and dusty winds from the northeast, originating from the Sahara and passing over West Africa into the Gulf of Guinea. Even though the weather is relatively hot, residents usually lock their windows and doors to avert dust from entering their rooms during this period.

There was no known study conducted in Ghana specifically the study area, that takes into account the seasonal radon disparities, correction factors, and radon mapping. Therefore, this study measures indoor radon seasonal variation in rainy, dry as well as harmattan seasons, generates spatial mapping and also determines correction factors for the studied location. This is with the view of identifying the weather season with higher radon exposure and locating the areas with elevated radon levels that are associated with this season to prevent or decrease the effect of radon gas exposures on the dwellers.

## Materials and methods

### Summary of methodology

**Fig 1:**
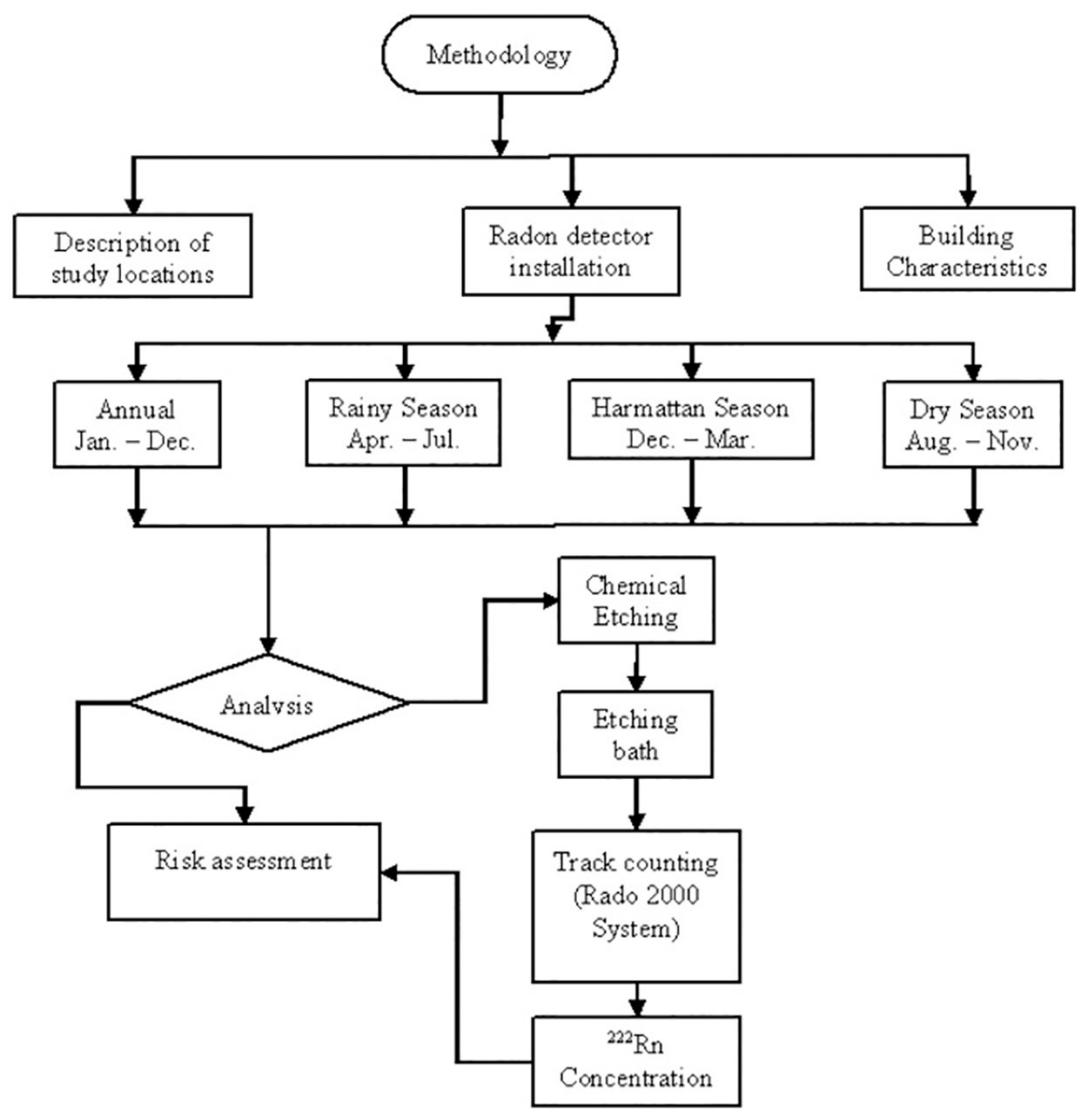


Fig 1 shows the flowchart of the methodology used for the measurement and analysis of the indoor radon data for this study. A total of 120 dwellings and 480 CR-39 detectors were used to measure indoor radon concentrations during rainy, harmattan and dry weather seasons, and one-year measurement.

### Description of the study location

**Fig 2:**
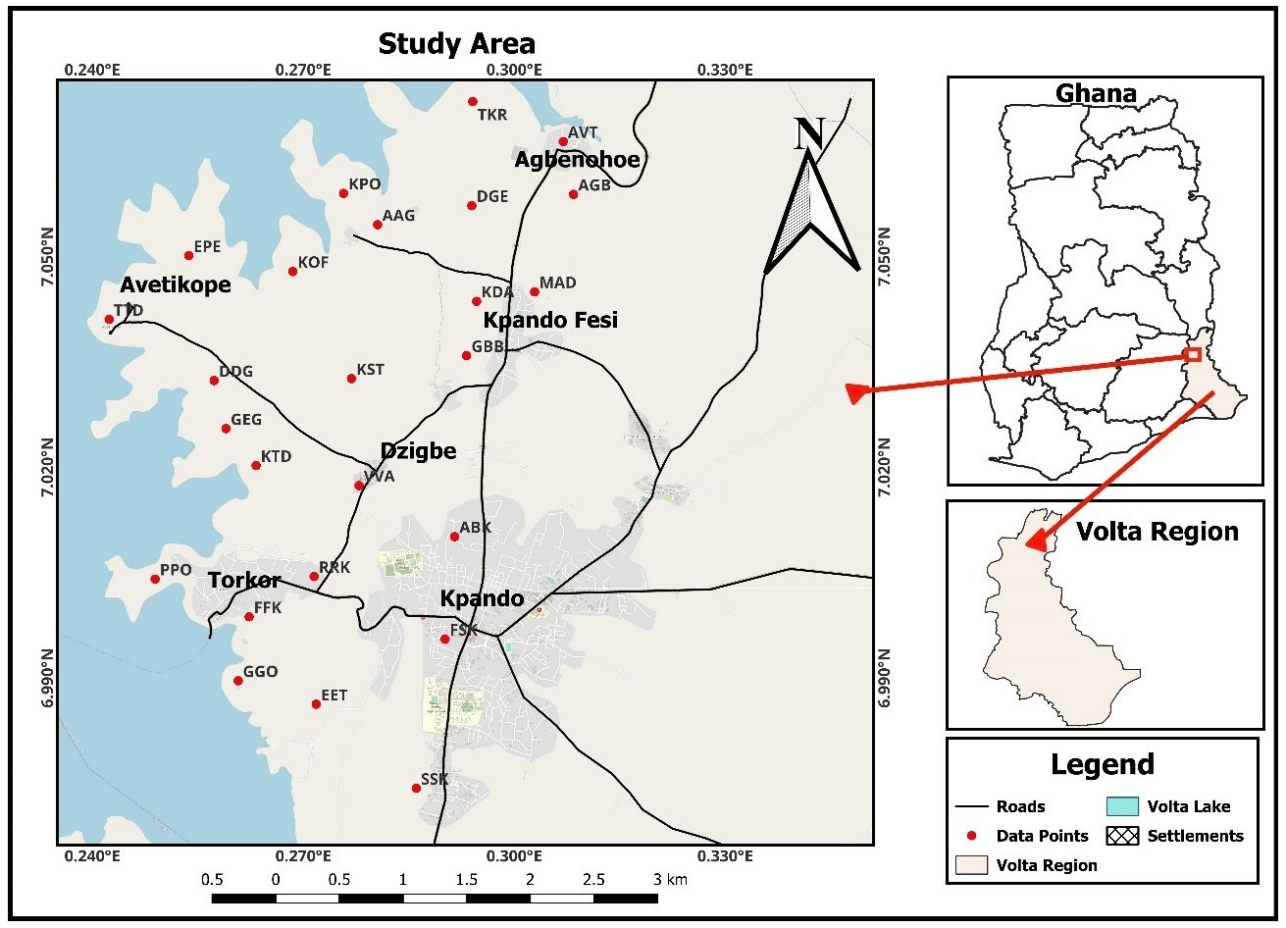


The study locations selected are situated within the Kpando Municipality of the Volta Region located in the southern part of Ghana as shown in Fig 2. The mean annual temperature of the Municipal is 27 °C with the mean daily temperature varying from 22 to 33 °C. The overall land area of the municipal is around 820 square kilometres, which accounts for 4.5% of the Volta Region, with the Volta Lake covering approximately 12% of the land area. The Volta Lake, which runs along over 80 kilometres of the shoreline, serves as the dividing line on the western side of the territory. The municipal area is bounded to the north and east by Biakoye District and Afadzato South District as well as by North Dayi District [22].

### Characteristics of building materials

The major building materials used in the construction of dwellings in the study locations is clay with cement walls and concrete floors, clay with cement walls and clay floors, and sandcrete and concrete blocks. Most of the dwellings are clay with cement walls and clays, due to the low daily income. Many of the populaces are fishermen and farmers who engage in small-scale farming.

### Radon detector installation and analysis

This study utilizes CR-39 (SSNTDs) detectors to measure indoor radon concentration on a yearly and seasonal basis. About 480 CR-39 detectors were exposed to measure indoor radon concentrations within the study location (R_S_: July to October; D_S_: March to June and H_S_: November to February). One hundred and twenty (120) homes in various locations within Kpando Municipality were used. The CR-39 detectors were exposed within the rooms for a period of a year and for 4 months each in the D_S_, R_S_, and H_S_. The CR-39 detectors were exposed in bedrooms at a height of 1.0 to 1.5 m from the floor and also about 0.5 m or more and 0.15 m away from the walls and from any other things, respectively [11]. After the exposure period, the exposed radon detectors were removed and sent to the Radiation Protection Institute, Ghana Atomic Energy Commission (RPI/GAEC) for counting and analysis [27].

### Chemical etching and analysis

The exposed CR-39 detectors were detached and etched in 6.25 N in a prepared solution of 4000 ml of distilled water comprising 1000 g pellet at 90 °C for four hours and thirty minutes to magnify the tracks from the alpha particles that form on the film detector from the radon daughters on exposed detectors. Finally, the film was dry for four days after etching. The tracks established on the CR-39 film were evaluated and examined counted in 144 fields with an optical microscope consisting of a 40 × magnification objective lens [10, 27]. Tracks density remained on detector films were used to calculate radon concentration, C_Rn_ as expressed in Eq (1):

$$C_{Rn}\left(Bqm^{-3}\right)=\frac{\rho }{\varepsilon t}$$ (1)

where ε = calibration factor of detector (track/cm^2^d/(Bq/m^3^), ρ = measured surface density of tracks (tracks/cm^2^) and t = exposure time.

### Annual effective dose (A_E_)

A_E_ to individuals within the studied areas associated with exposure to indoor radon gas was estimated based on Eq (2) recommended by UNSCEAR [1]:

$$A_{E}=C_{Rn}\times I_{of}\times D_{cf}\times T_{h}$$ (2)

Where *I*_*of*_: indoor occupancy factor (0.8), C_Rn_: radon concentration, *T*_*h*_: time in hours per year (8760h/y) and *D*_*Cf*_: dose conversion factor (9.0nSv/h) [1].

### Spatial analysis

The mapping of the radon concentration in dwellings was done using the Inverse Distance Weighting interpolation approach. The map was generated by depicting the radon concentration spatial distribution for dwellings. The geological spatial map for radon data within the study dwellings was done utilizing ArcGIS geostatistical software version 10.7.1 from GDi Esri Hungary Ltd., Budapest, Hungary [28]. The map was generated over a grid with dimensions of 100 m by 100 m [8]. The radon levels of radon distributions were described in the form of intensity using the red colour from light to deep representing the lowest and highest respectively within the Kpando district.

### Effect of building characteristics on indoor radon

Radon effect on building characteristics within the study locations was determined by differences in construction materials: clay, concrete, wood, and sandcrete as well as the years of the dwelling’s establishment. The 120 dwellings with similar factors such as building materials, windows, and doors, were used as a factor to determine the ventilation rate within 26 studied areas. Dwelling with only one window and a door is considered poor ventilation while double windows and doors are classified as well-ventilated rooms.

### Descriptive statistical estimation

Statistical evaluation associated with radon data was assessed in the form of Pearson linear correlation coefficient, median, geometric mean, standard deviation, arithmetic mean, mode, minimum, and maximum range values, using SPSS version 26 and OriginPro 2022 version. This is to determine the uncertainty and relationship between the variations of the different seasons and to estimate its dependence on annual radon concentration within the study environment [29].

## Results and discussions

### Indoor radon distribution

**Fig 3:**
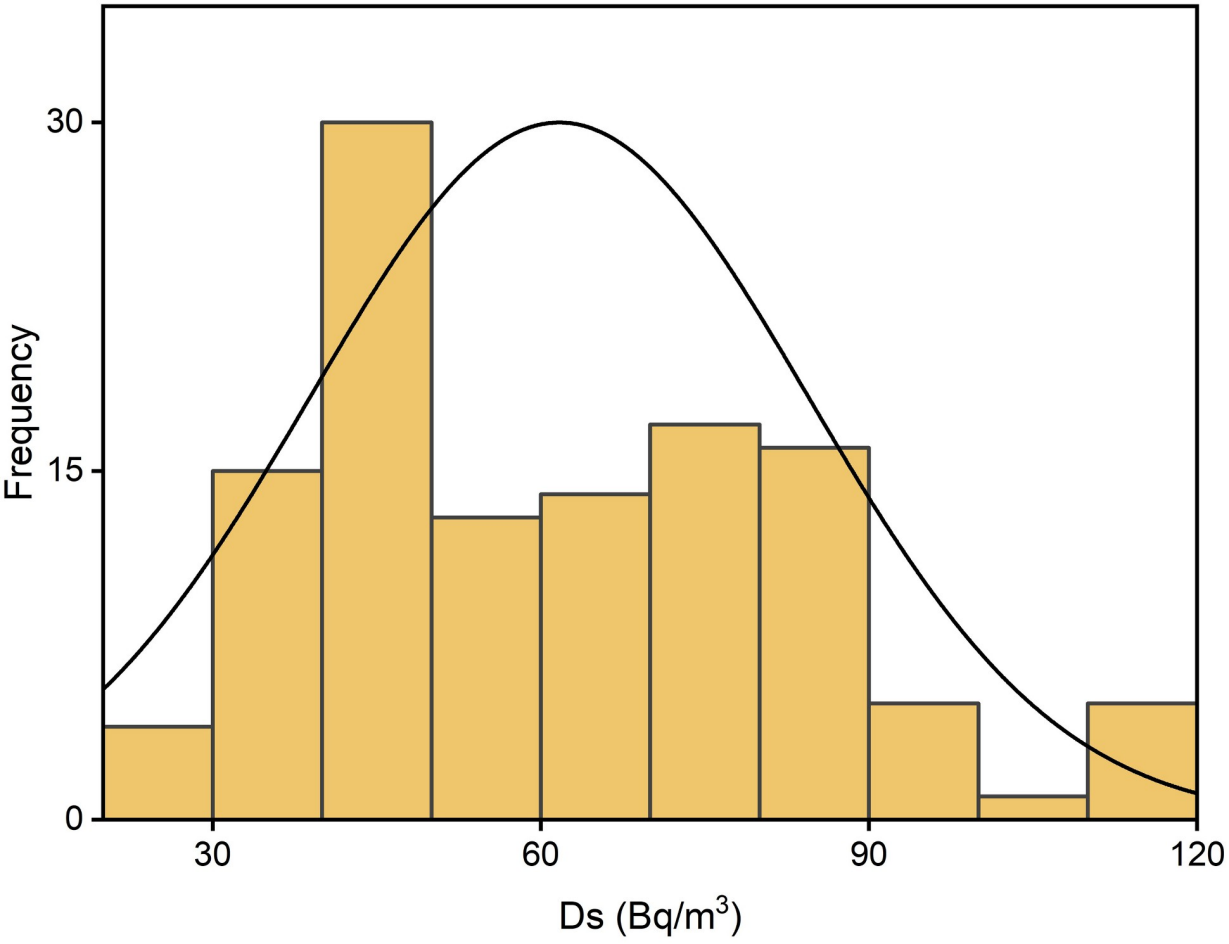


**Fig 4:**
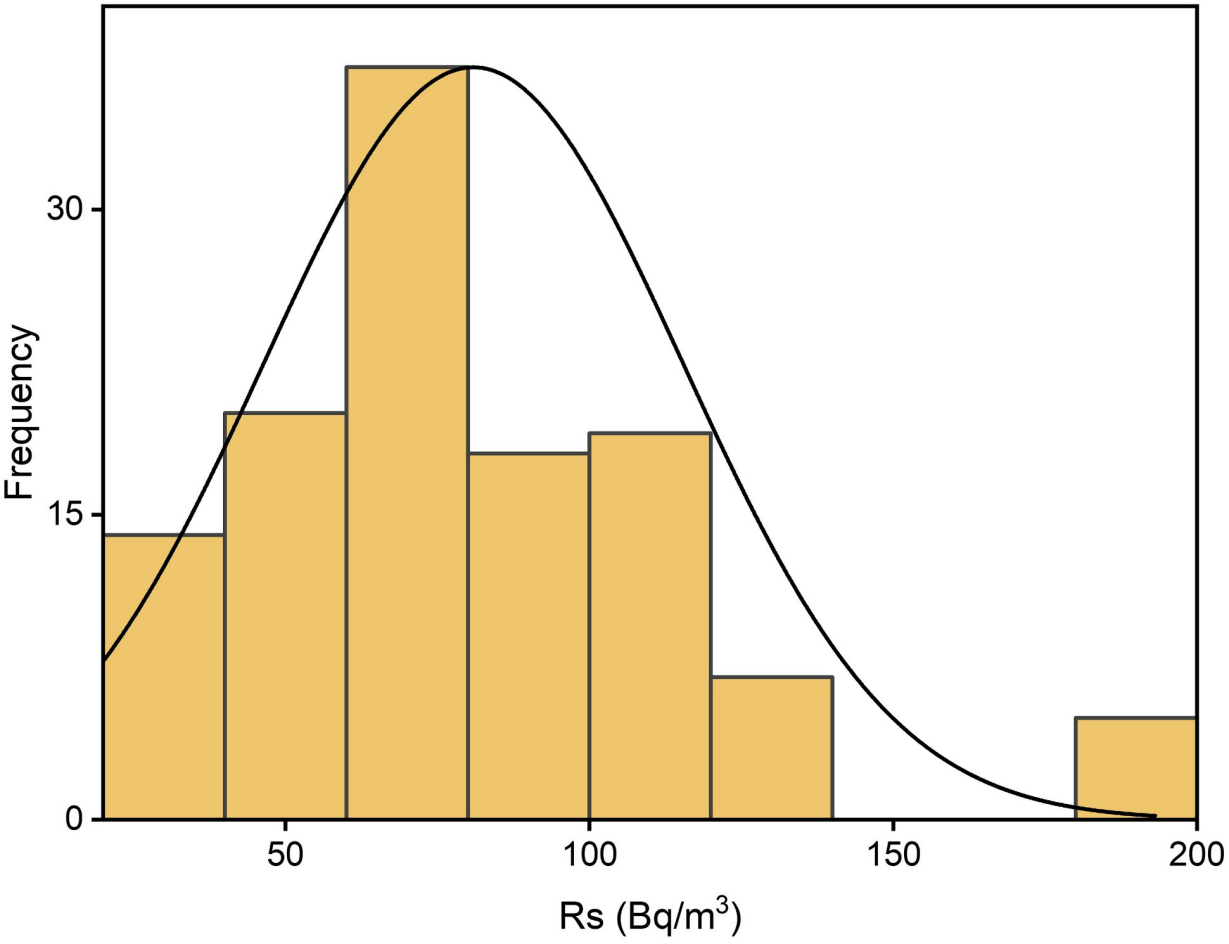


**Fig 5:**
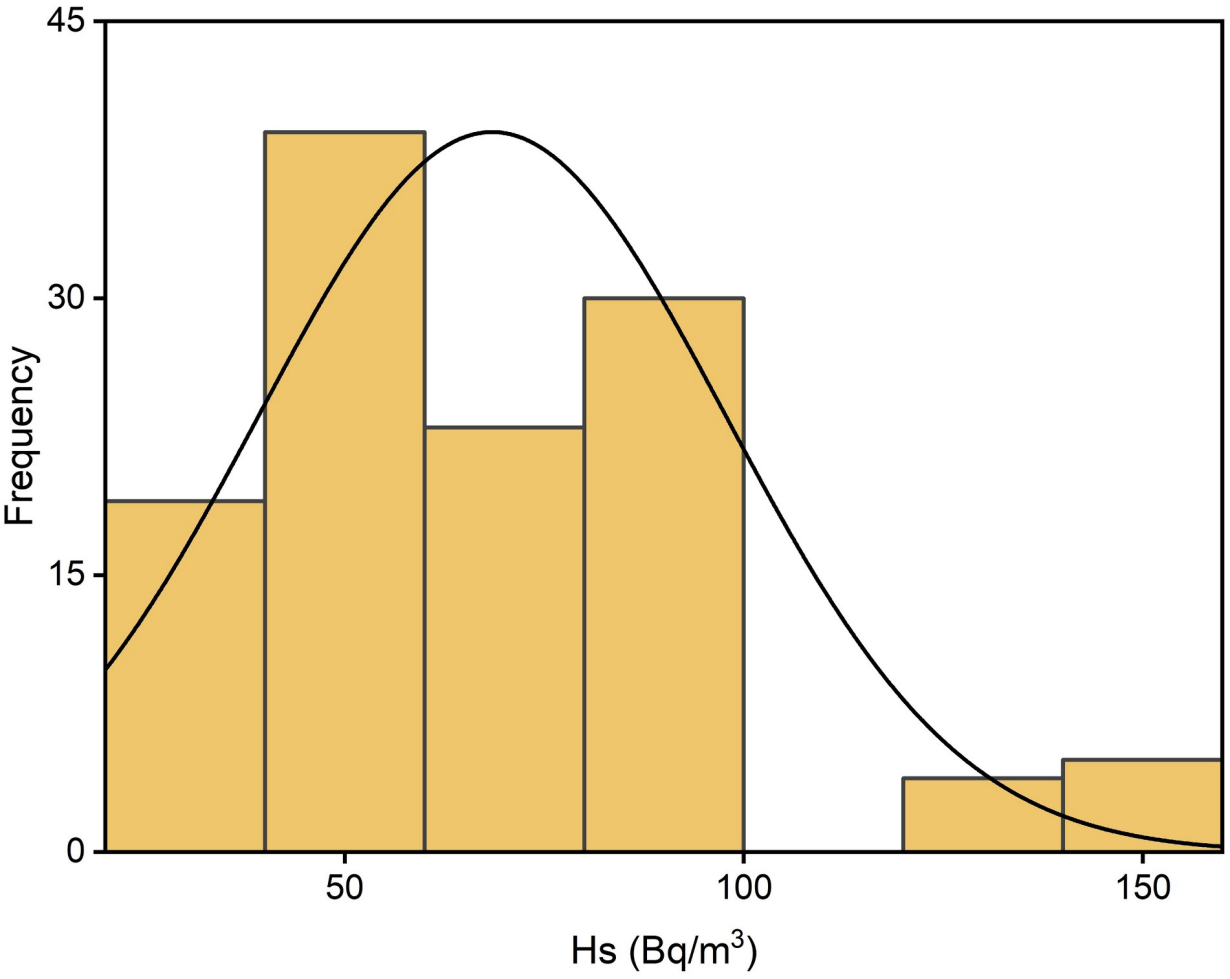


**Fig 6:**
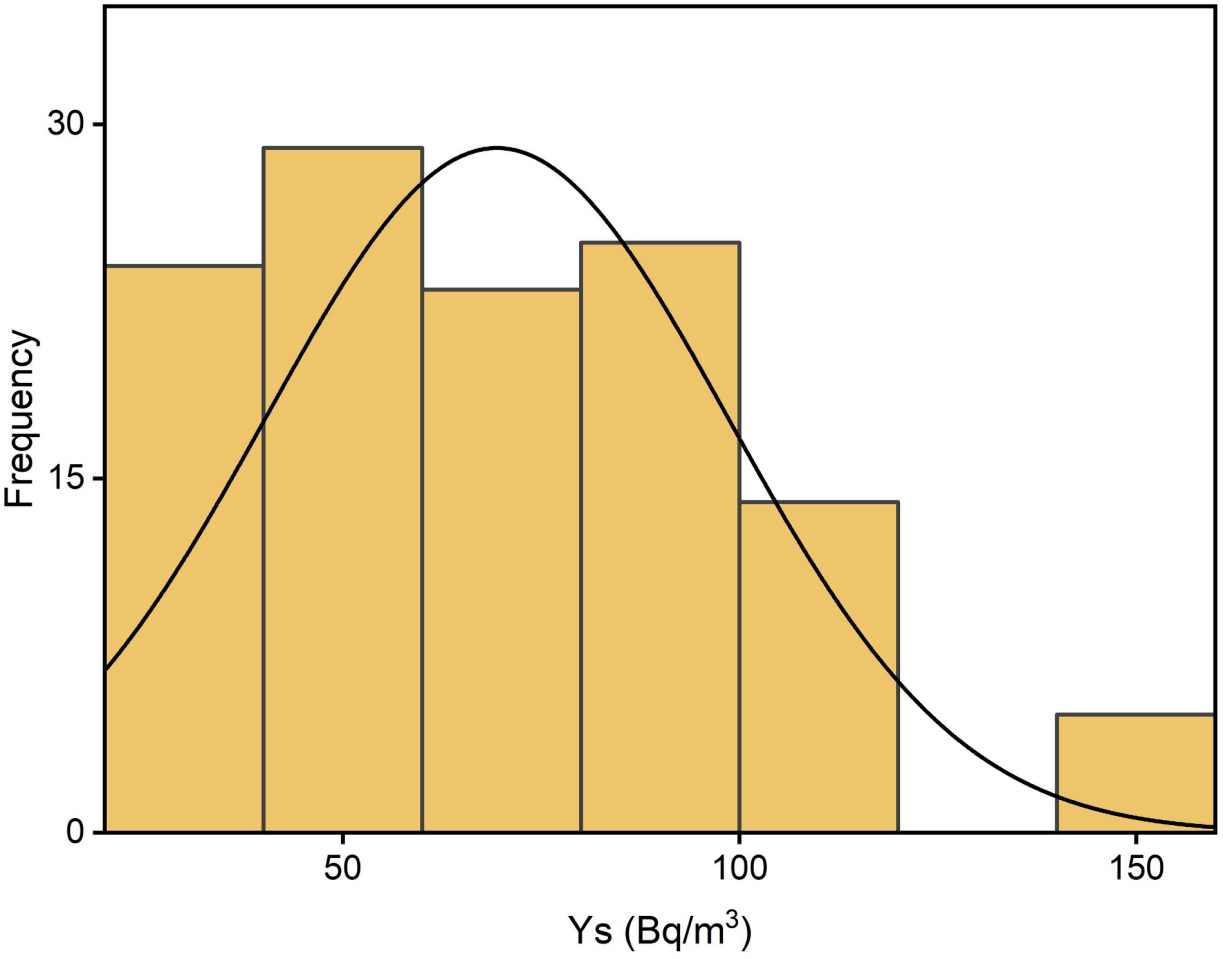


**Fig 7:**
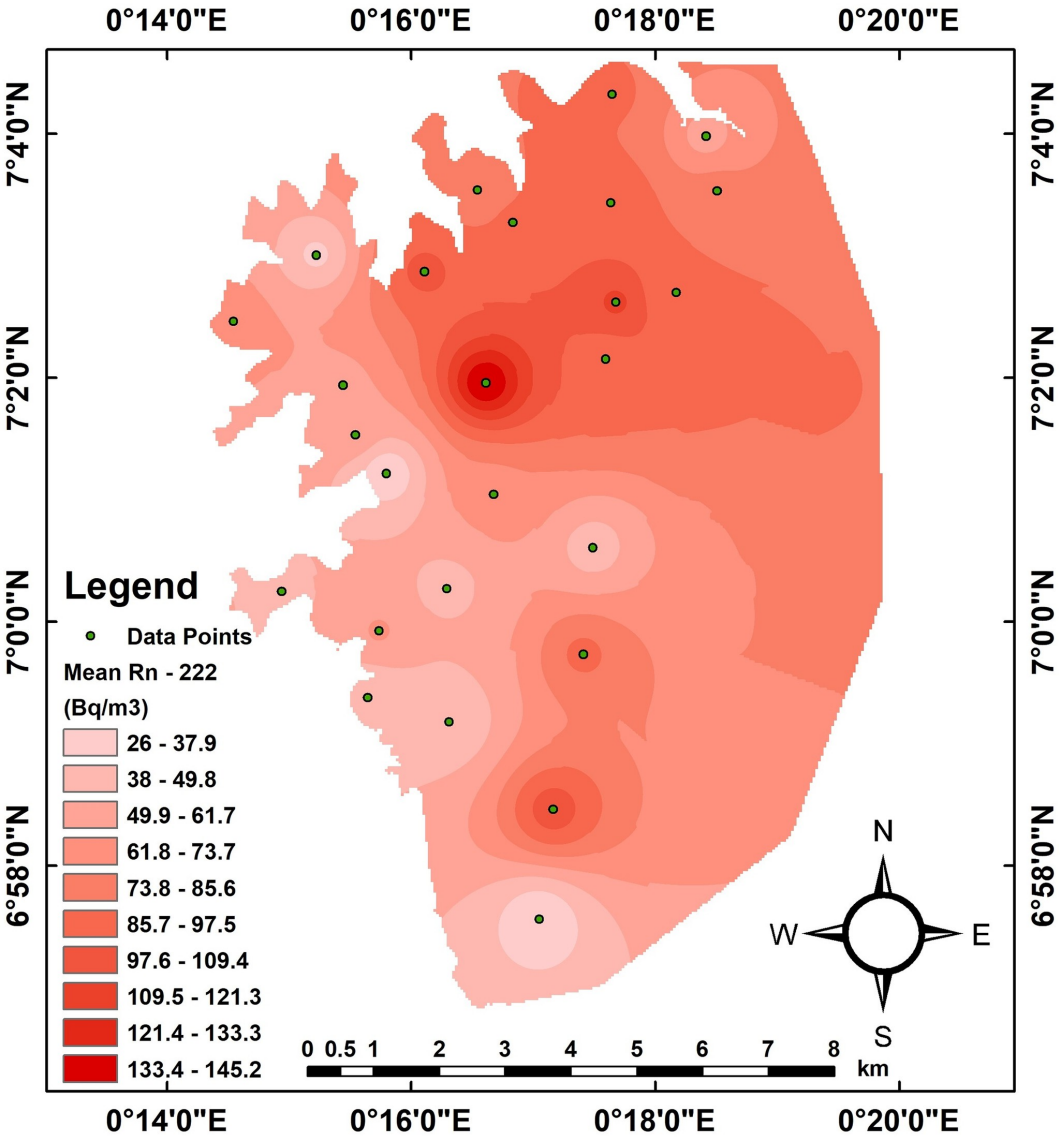


Radon distribution levels measured in the Kpando Municipality during three seasons and annually are depicted in Figs 3–6. The frequency distribution of all the graphs for the seasons and annual radon data shows that indoor radon in the studied locations is not normally distributed but rather appears to be log-normal as reported by other studies [8, 11, 29, 30]. The nonexistence of a normal distribution for indoor radon data could result from various factors, including different buildings and their age, occupant behaviour, building materials, the type of soil/rock beneath the buildings, ventilation conditions, as well as environmental and seasonal factors. The spatial distribution map also indicates that indoor radon concentration within the Kpando district is not uniformly distributed throughout the study dwellings. Radon exposure levels or intensities received vary across all the study communities. The intensity of the indoor radon exposure levels is represented by colours, with the higher levels indicated in deep red and lower exposure levels represented in light red. The radon mapping was plotted with various calculated mean values within each grid section, as shown in Fig 7.

The non-uniform distribution observed within the study locality can be attributed to varying radium concentrations across all dwelling locations [3, 8]. The measured radon concentration within the Kpando district, in 120 different dwellings, is presented in the form of a range, mean, median, mode, etc. The study determines Y_S_, R_S_, H_S_, and D_S_ and the results are tabulated in Tables 1 and 2. Results for indoor radon concentration for the Y_S_ values range from 24.8 to 190.1 Bqm^-3^ with a mean of 70.4 ± 1.6 Bqm^-3^, while seasonal indoor radon concentrations were also presented as range and mean values: 24.8 to 112.9 with a mean of 61.7 ± 2.1 Bqm^-3^, 30.8 to 151.4 Bq/m^3^ with a mean of 68.5 ± 2.7 Bq/m^3^, and 32.3 to 190.1 Bqm^-3^ with a mean of 80.9 ± 3.2 Bqm^-3^ for D_S_, H_S_ and R_S_ respectively as presented in Table 2.

**Table 1:** Indoor radon concentrations during three seasons and annual.

EL	ND	Mean seasonal and annual indoor radon concentrations (Bq/m^3^)				A_E_ (mSv/y)
		D_S_	R_S_	H_S_	Y_S_	
AGB	5	69.1 ± 0.2	88.5 ± 0.5	73.3 ± 0.3	76.0 ± 0.5	1.9 ± 0.0
AVT	5	48.9 ± 0.4	75.6 ± 0.5	54.5 ± 1.0	56.9 ± 0.5	1.5 ± 0.0
TKR	3	56.0 ± 0.3	71.1 ± 0.2	65.0 ± 0.3	88.0 ± 0.4	2.1 ± 0.0
DGE	3	89.6 ± 0.3	102.0 ± 0.3	90.9 ± 0.5	95.9 ± 0.9	2.4 ± 0.0
KPO	3	75.6 ± 0.3	88.4 ± 0.5	83.0 ± 0.3	78.0 ± 0.6	1.9 ± 0.0
KDA	5	65.4 ± 0.4	110.2 ± 0.4	67.9 ± 0.3	112.6 ± 0.8	2.8 ± 0.1
KOF	3	99.7 ± 0.2	121.1 ± 0.2	129.0 ± 0.4	101.4 ± 0.3	2.6 ± 0.0
TTD	3	67.1 ± 1.0	79.1 ± 0.2	65.7 ± 0.6	67.2 ± 0.4	1.7 ± 0.1
GBB	3	85.1 ± 0.2	99.3 ± 0.7	88.6 ± 0.6	91.1 ± 0.7	2.3 ± 0.0
AAG	3	79.7 ± 0.6	121.0 ± 0.9	92.0 ± 0.3	85.9 ± 0.6	2.2 ± 0.0
DDG	3	53.5 ± 0.8	66.0 ± 0.6	56.7 ± 0.5	58.8 ± 0.8	1.5 ± 0.0
KTD	5	30.3 ± 0.4	34.0 ± 0.2	34.0 ± 0.3	25.9 ± 0.4	0.7 ± 0.1
VVA	5	44.0 ± 0.1	65.9 ± 0.2	54.9 ± 0.3	69.5 ± 0.4	1.8 ± 0.0
RRK	5	32.7 ± 0.2	56.1 ± 0.5	40.5 ± 0.4	44.7 ± 0.4	1.2 ± 0.1
PPO	5	58.7 ± 0.3	67.9 ± 0.1	50.7 ± 0.3	47.8 ± 0.7	1.3 ± 0.1
FFK	5	70.7 ± 0.6	78.4 ± 0.4	73.1 ± 0.2	63.1 ± 0.7	1.6 ± 0.0
GGO	5	44.8 ± 0.2	37.6 ± 0.5	32.6 ± 0.5	37.7 ± 0.5	1.0 ± 0.0
KST	5	111.4 ± 1.0	117.9 ± 1.2	99.1 ± 0.7	103.2 ± 0.5	2.6 ± 0.0
SSK	5	88.6 ± 0.5	188.9 ± 0.9	150.7 ± 0.2	144.4 ± 0.6	3.8 ± 0.1
FSK	3	90.4 ± 1.4	109.4 ± 0.9	96.7 ± 0.6	88.9 ± 0.3	2.5 ± 0.0
ABK	5	75.0 ± 0.2	93.2 ± 0.3	87.9 ± 0.3	43.0 ± 0.4	1.1 ± 0.0
GEG	5	43.2 ± 1.5	63.8 ± 0.2	55.0 ± 0.3	55.1 ± 0.8	1.3 ± 0.1
MAD	5	40.4 ± 0.3	56.3 ± 1.1	46.8 ± 0.2	85.8 ± 0.8	2.2 ± 0.0
EPE	5	36.2 ± 1.9	45.2 ± 1.2	35.8 ± 0.9	35.3 ± 0.7	0.9 ± 0.0
EET	5	43.5 ± 0.3	50.2 ± 0.2	47.8 ± 0.2	38.2 ± 0.2	1.0 ± 0.0
DEM	3	25.6 ± 0.8	33.0 ± 1.1	31.4 ± 0.5	29.1 ± 0.3	0.7 ± 0.0

EL = Exposure location, ND = Number of dwellings

**Table 2:** Basic statistics for the study of radon parameters.

Statistics	Seasonal indoor radon parameters (Bq/m^3^)				Overall data (Bq/m^3^)	A_E_ (mSv/y)
	D_S_	R_S_	H_S_	Y_S_		
Mean	61.7	80.9	68.5	69.4	70.4	1.8
SD	2.1	3.2	2.7	2.7	1.6	0.1
GM	57.5	74.2	62.9	63.3	64.5	1.6
Median	58.8	75.8	64.9	67.1	66.2	1.7
Mode	85.0	110.0	50.9	38.0	88.9	1.0
Minimum	24.8	32.3	30.8	25.2	24.8	0.6
Maximum	112.9	190.1	151.4	145.2	190.1	4.0

SD = Standard deviation, GM = Geometric mean and A_E_ = Annual effective dose (mSv/y)

Approximately, 21% of the total radon data, including R_S_, H_S_, and D_S_ recorded values exceeding the WHO [2] reference level of 100 Bqm^-3^, with R_S_ having 11%, H_S_ having 7%, and D_S_ having 3% of seasonal radon data greater than the WHO reference level. The areas that recorded values exceeding the WHO [2] reference level are KDA, KOF, KST, and SSK, while the lowest value was found in DEM. The highest indoor radon values recorded in these areas were all located in densely populated areas with poor ventilation, which may be responsible for radon levels exceeding 100 Bq/m^3^ [2]. The higher percentage of radon recorded during R_S_ may be due to doors and windows being closed for longer periods compared to H_S_ and D_S_, leading to the buildup of radon gas originating from building materials and soil beneath the dwellings.

### Effect of building characteristics on indoor radon concentration

**Fig 8:**
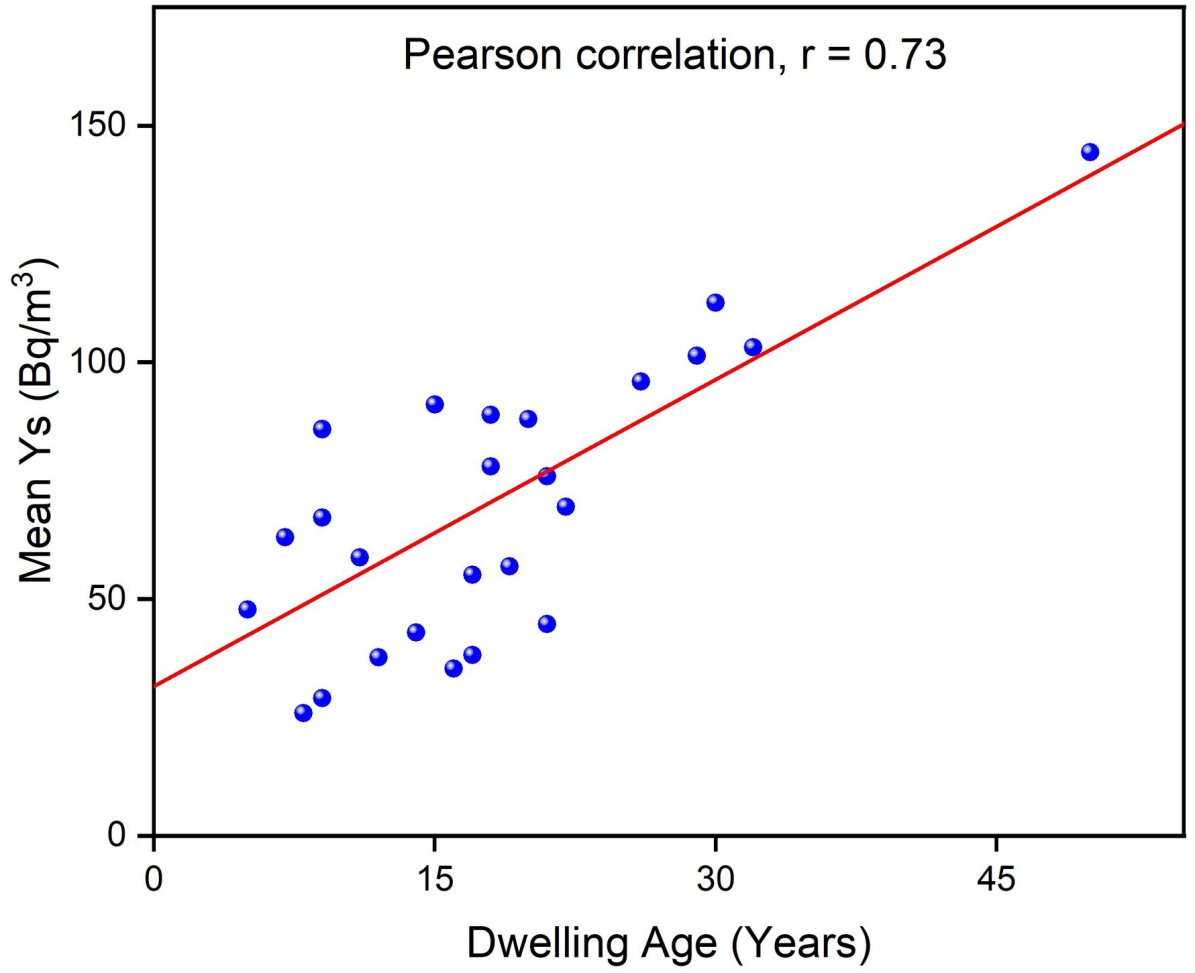


This study examined various building characteristics, including dwelling age, construction materials, and ventilation rate. The radon concentration data from this study, with dwelling age ranging from 5 to 50 years, revealed average values ranging from 15 to 89 Bq/m^3^. The lowest average value was recorded in dwellings less than 5 years old, while the highest value was obtained in dwellings aged 50 years. Pearson’s correlation analysis revealed a strong positive coefficient value of 0.73 between radon concentration and dwelling age. In other words, increasing dwelling age will lead to a decrease in indoor radon concentration as shown in Fig 8. This observation suggests that as buildings become old, cracks or gaps may develop under the foundations, and pipelines which may lead to an increase in radon emanation from construction materials and soil beneath the dwellings. These caused a buildup of radon gas in the rooms resulting in higher radon levels. These findings align with similar studies conducted by other researchers [31, 32].

**Fig 9:**
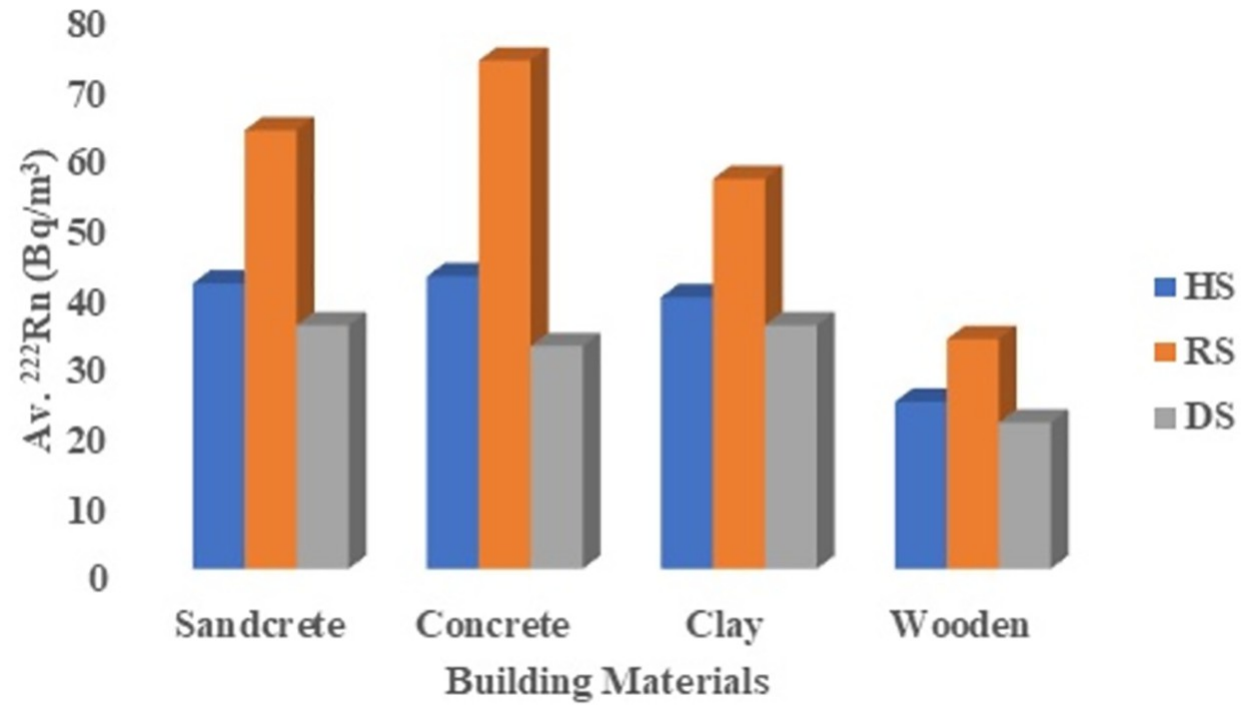


Indoor radon concentration associated with these dwellings made of different building materials has been assessed. Seasonal radon data revealed that concrete and wooden dwellings exhibited higher and lower radon levels in all seasons, as shown in Fig 9. The lower radon levels associated with wooden dwellings may be attributed to the absence of wood in soil or rocks, which are part of the Earth’s crust. Consequently, wooden structures contain a smaller amount of radium-226, which decays to produce radon alpha particles, resulting in lower radon levels compared to other construction materials. Additionally, the larger spaces between wooden structures promote continuous air exchange between the indoor and outdoor environment, thereby reducing the radon levels in the rooms. These findings are consistent with previous studies conducted in Ghana by Otoo [8, 11, 26].

### Seasonal radon variations

**Fig 10:**
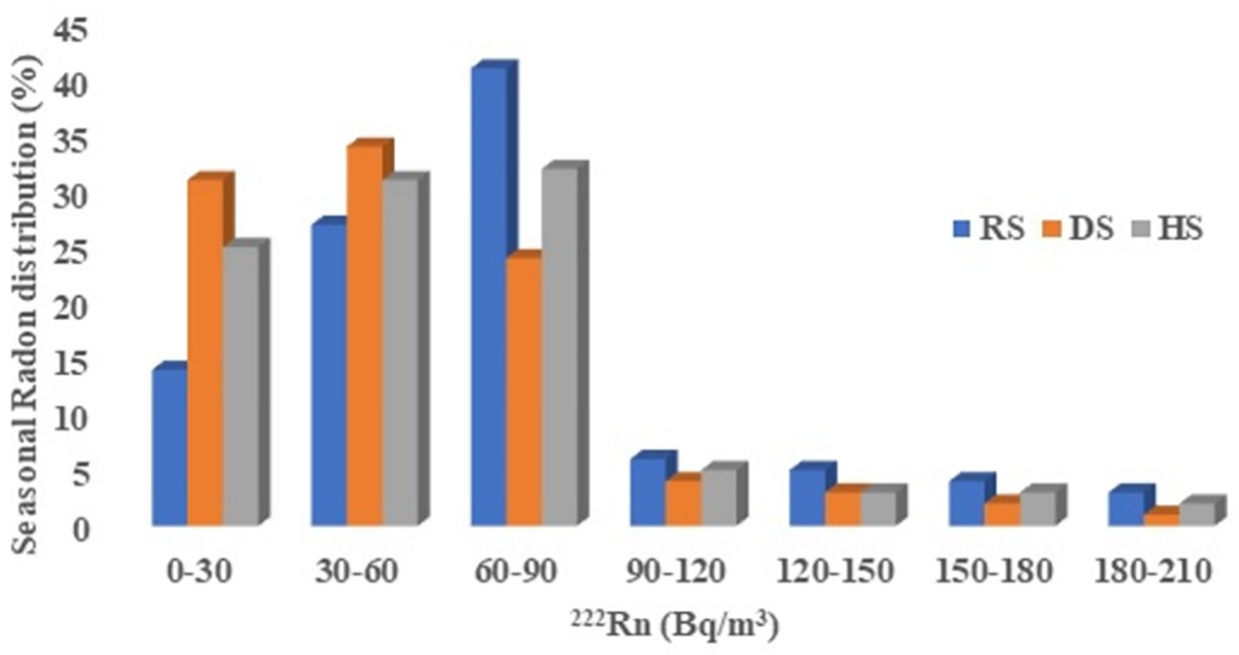


Seasonal variations in indoor radon levels indicated that Rs and Hs recorded the highest frequency distribution, with about 41% and 32%, falling within the range values of 60–90 Bq/m^3^. Additionally, about 34% of radon distribution data occurred within the range values of 30–60 Bq/m^3^, which represented the highest percentage for Ds. Dwellings exhibited the lowest frequency distribution for all the seasonal radon data, with the range of 180–210 Bqm^-3^ as shown in Fig 10. Across all seasonal radon data, approximately, 11%, 7%, and 3% exceeded the WHO [2] recommended reference value of 100 Bqm^-3^ for Rs, Hs and Ds values, respectively. The higher values associated with Rs might be due attributed to doors and windows within the rooms being kept shut during this period, preventing the circulation of air from the outside environment into the dwellings. This leads to poorly ventilated rooms and the buildup of radon concentration from construction materials and soil beneath the dwellings, which may explain the higher radon concentrations recorded within R_S_. These findings are consistent with some studies conducted in Ghana [8, 11, 13]. The higher values during Rs may also be caused by the surrounding soil being wet during rains, which pushes radon particles to emanate through the soil beneath the dwellings, thereby increasing the radon gas concentrations in the rooms.

Radon concentrations associated with Hs were found to be less than Rs but higher than during Ds, as depicted in Fig 10. This difference may be due to the cool, dry wind which frequently carrying large amounts of dust indoors during H_S_, forcing residents to close their windows and doors. This is done to prevent dust from entering the rooms, causing radon concentration to accumulate to levels higher than during D_S_, where windows and doors are kept open to mitigate hot weather conditions.

### Pearson correlation analysis

**Fig 11:**
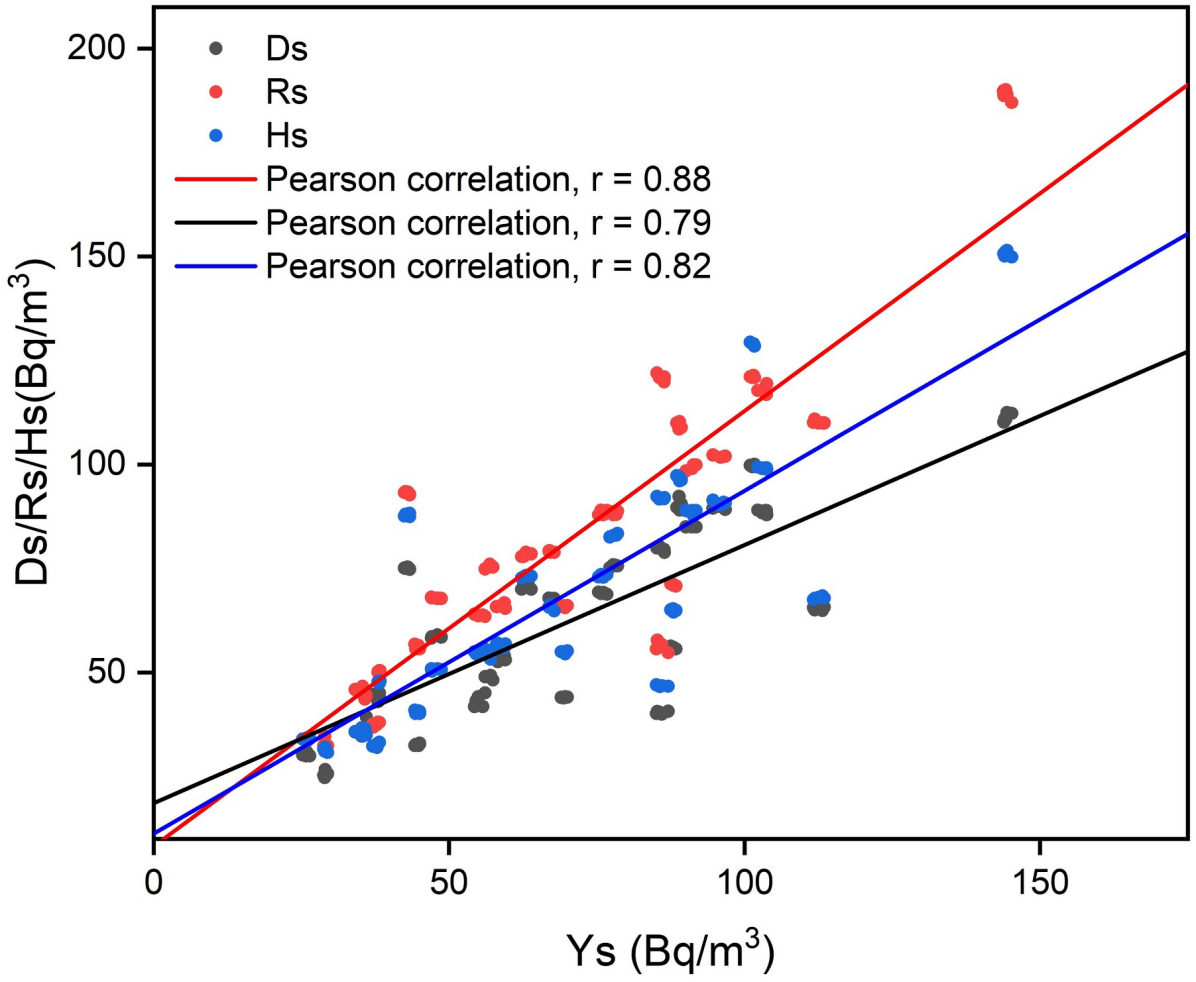


The relationship between the study data was determined using Pearson’s statistical tool to explore the connection between seasonal indoor radon and annual radon concentration. This analysis was also conducted to understand the influence of seasonal variation conditions on indoor radon concentration, as illustrated in Fig 11. Positive correlations were obtained for all the correlations analyzed between the annual and three weather seasons: R_S_, H_S_, and D_S_. The maximum positive correlation value was observed between Ys and Rs (0.88), and the minimum value was recorded between the correlation of Ys and Ds (0.79), while Hs with Ys had a correlation value greater than Ds but less than Rs and Ys, as depicted in Fig 10. The variations in the correlation coefficient values for the weather seasons may be attributed to weather conditions, occupant behaviour in terms of closing doors and windows, difference in building styles, as well as variation in radium-226 concentration within the study location.

The highest and lowest values observed within Rs and Ds with Ys can be attributed to the similar factors that accounted for the largest and smallest mean seasonal radon values occurring within Rs and Ds in the dwellings, as stated in Fig 10. These results are consistent with studies conducted in the Greater Accra region of Ghana [8, 12].

### Seasonal correction factors (S_CF_)

The seasonal correction factor (S_CF_), a numerical multiplier used to convert short-term radon concentration measurements to annual average concentrations, was computed and tabulated in Table 3. From Table 3, the seasonal correction factor varied from 0.9 to 1.2. The maximum S_CF_ was obtained during Rs, while the minimum S_CF_ value was recorded during Ds. The S_CF_ during Hs was found to be less than that of Rs and greater than Ds value. The difference in these correction factors may be attributed to the same conditions that contributed to Pearson’s correlation values and the variations recorded within the seasonal radon concentrations existing in the rooms, as shown in Fig 10.

**Table 3:** Seasonal radon correction factor (S_CF_).

Seasons	S_CF_
Dry	0.9
Rainy	1.2
Harmattan	1.0

### Annual effective doses (A_E_)

Annual effective doses estimated due to the mean radon concentration received by inhabitants were presented in Tables 1 and 2. The calculated A_E_ from the study locations from Table 1 ranges from 0.6 to 4.0 mSv/y, with an associated overall arithmetic mean value of 1.8 ± 0.05 mSv/y. The geometric mean is 1.6 mSv/y, the mode is 1.0 mSv/y, and the median is 1.7 mSv/y. Table 2 shows the mean A_E_ with respect to the exposure locations, with calculated values ranging from 0.7 ± 0.1 (KTD) to 3.8 ± 0.1 mSv/y (SSK). In all the exposure locations, the mean values are less than the value recommended by the International Commission on Radiological Protection (ICRP) [33]. However, at DGE, KDA, KOF, KST, SSK, and FSK, the mean values were found to be greater than the 2.4 mSv/y proposed by the United Nations Scientific Committee on the Effects of Atomic Radiation (UNSCEAR) [1]. The overall arithmetic mean obtained from this study was 2.3 and 1.8 times smaller than the lower limit of the action level and world average values from ICRP and UNSCEAR, respectively, as shown in Table 2.

### Limitations

This study is subject to some limitations. The number of premises in which the radon measurements were performed was small, and the seasonal indoor radon level variations were determined in residential buildings only which have no air-conditioner system. The results of this study can be influenced by changing weather conditions and human behaviour, potentially leading to inaccuracies. Therefore, future research is needed to analyze the relationships between radon concentrations and meteorological parameters in office and school buildings.

## Conclusion

The study investigated seasonal and annual indoor radon, correction factors, and effects of dwelling characteristics on indoor radon levels in Kpando Municipality. The radon data for both the annual and the three weather seasons were found to be non-uniformly distributed. The highest and lowest seasonal radon data were noted during the rainy and dry periods, respectively. A positive Pearson’s correlation coefficient and seasonal correction factors indicated the effects of seasonality on indoor radon concentration variations. Building materials and dwelling age were found to have significant effects on radon concentration in rooms. Approximately, 21% of the radon data obtained in this study exceeded the reference levels recommended by WHO [2], with most locations having radon values far below the proposed lower ranged values of 200 to 600 Bq/m^3^ by the ICRP [34]. Spatial map of radon levels was also produced to highlight areas of low or high radon concentrations. The regulatory authorities responsible for monitoring and managing radon levels will see the map generated in this study to be a valuable resource for a proactively preventing and mitigating radon in residential environments. Additionally, these findings may support the integration of construction methods designed to withstand radon infiltration. Nevertheless, for the study dwellings with radon concentrations exceeding the WHO reference level, it is strongly recommended to ensure proper air flow circulation between the indoor and outdoor environment. This is essential to reduce or prevent any risk of future lung cancer due to prolong or elevated exposure to radon particles. The annual effective dose was found to be below the proposed dose limit set by the ICRP [34].

## Supporting information

S1 File Minimal data set. (DOCX)
