# Supplementary material for: Assessment of indoor radon distribution and seasonal variation within the Kpando Municipality of Volta Region, Ghana
Source: PLoS One. 2024 Feb 27;19(2):e0299072. doi: 10.1371/journal.pone.0299072 (PMC10898764; doi:10.1371/journal.pone.0299072)
Supplement: S1 File — (DOCX) [file pone.0299072.s001.docx]

| Dry season | | | | | | | |
| --- | --- | --- | --- | --- | --- | --- | --- |
| EL | GPRS | | Number of Dwellings in exposure location | | | | |
|  | Longitude | Latitude | R1 | R2 | R3 | R4 | R5 |
| AGB | 7.098616 | 0.277741 | 68.8 | 69.3 | 69.2 | 69.0 | 69.1 |
| AVT | 7.088344 | 0.280118 | 48.9 | 49.0 | 49.3 | 49.1 | 48.2 |
| TKR | 7.088187 | 0.272883 | 56.0 | 55.9 | 56.3 | 55.7 |  |
| DGE | 7.091226 | 0.263588 | 89.2 | 89.9 | 89.9 | 89.5 |  |
| KPO | 7.084466 | 0.255666 | 75.6 | 75.9 | 75.2 | 75.7 |  |
| KDA | 7.067490 | 0.261050 | 65.0 | 65.6 | 64.9 | 65.7 | 65.6 |
| KOF | 7.067070 | 0.276260 | 99.5 | 100.0 | 99.8 | 99.5 |  |
| TTD | 7.061200 | 0.289890 | 67.8 | 67.9 | 67.0 | 65.8 |  |
| GBB | 7.057950 | 0.250910 | 85.3 | 85.0 | 85.0 | 85.0 |  |
| AAG | 7.060880 | 0.237290 | 78.9 | 79.6 | 80.3 | 79.9 |  |
| DDG | 7.049880 | 0.238450 | 52.6 | 54.2 | 54.0 | 53.0 |  |
| KTD | 7.052080 | 0.272250 | 30.2 | 31.0 | 30.5 | 30.0 | 30.0 |
| VVA | 7.044420 | 0.270770 | 44.2 | 43.9 | 44.0 | 43.9 | 44.1 |
| RRK | 7.029540 | 0.242780 | 32.4 | 33.0 | 32.5 | 32.7 | 32.8 |
| PPO | 7.018850 | 0.249220 | 58.6 | 59.0 | 58.5 | 58.9 | 58.3 |
| FFK | 7.004800 | 0.271830 | 70.0 | 71.3 | 70.9 | 71.1 | 70.0 |
| GGO | 7.025450 | 0.222080 | 44.5 | 45.0 | 44.7 | 45.1 | 44.9 |
| KST | 7.015280 | 0.223030 | 112.5 | 110.2 | 111.0 | 112.3 | 110.9 |
| SSK | 7.003960 | 0.230740 | 88.9 | 89.0 | 88.8 | 87.9 | 88.4 |
| FSK | 7.009200 | 0.245840 | 89.1 | 92.3 | 90.5 | 89.7 |  |
| ABK | 7.001650 | 0.246370 | 75.1 | 75.0 | 74.8 | 74.9 | 75.3 |
| GEG | 6.989070 | 0.249860 | 41.8 | 44.2 | 43.2 | 41.8 | 45.1 |
| MAD | 6.983310 | 0.271510 | 40.7 | 40.1 | 40.6 | 40.4 | 40.0 |
| EPE | 6.962130 | 0.246050 | 36.7 | 34.7 | 34.7 | 35.7 | 39.3 |
| EET | 6.953950 | 0.245950 | 43.2 | 43.6 | 43.7 | 43.0 | 43.8 |
| DEM | 6.954790 | 0.261480 | 24.8 | 25.7 | 26.7 | 25.3 |  |

| Rainy season | | | | | | | |
| --- | --- | --- | --- | --- | --- | --- | --- |
| EL | GPRS | | Number of Dwellings in exposure location | | | | |
|  | Longitude | Latitude | R1 | R2 | R3 | R4 | R5 |
| AGB | 7.098616 | 0.277741 | 88.9 | 87.9 | 87.9 | 89 | 88.7 |
| AVT | 7.088344 | 0.280118 | 75.8 | 74.9 | 75.8 | 76 | 75.3 |
| TKR | 7.088187 | 0.272883 | 70.9 | 71.2 | 71.3 | 70.8 |  |
| DGE | 7.091226 | 0.263588 | 102 | 101.9 | 101.7 | 102.3 |  |
| KPO | 7.084466 | 0.255666 | 88.8 | 87.9 | 88.7 | 88 |  |
| KDA | 7.067490 | 0.261050 | 110.9 | 110 | 110 | 110 | 110.1 |
| KOF | 7.067070 | 0.276260 | 121.4 | 120.9 | 121.1 | 120.9 |  |
| TTD | 7.061200 | 0.289890 | 78.9 | 79.2 | 78.9 | 79.3 |  |
| GBB | 7.057950 | 0.250910 | 99.9 | 99.9 | 99 | 98.4 |  |
| AAG | 7.060880 | 0.237290 | 121 | 119.9 | 120.9 | 122 |  |
| DDG | 7.049880 | 0.238450 | 65.9 | 65.8 | 66.8 | 65.4 |  |
| KTD | 7.052080 | 0.272250 | 34 | 33.9 | 33.7 | 34.3 | 34.1 |
| VVA | 7.044420 | 0.270770 | 65.6 | 66 | 65.9 | 66 | 66.1 |
| RRK | 7.029540 | 0.242780 | 56.5 | 55.8 | 56.8 | 55.7 | 55.7 |
| PPO | 7.018850 | 0.249220 | 67.9 | 67.9 | 67.8 | 67.8 | 68.1 |
| FFK | 7.004800 | 0.271830 | 77.9 | 77.9 | 78.9 | 78.6 | 78.5 |
| GGO | 7.025450 | 0.222080 | 37.5 | 37.5 | 36.9 | 38 | 38.1 |
| KST | 7.015280 | 0.223030 | 188.9 | 188.7 | 189.6 | 187 | 190.10 |
| SSK | 7.003960 | 0.230740 | 116.8 | 117.8 | 117.9 | 119.4 | 117.80 |
| FSK | 7.009200 | 0.245840 | 110.3 | 108.5 | 108.9 | 110 |  |
| ABK | 7.001650 | 0.246370 | 93.3 | 93.5 | 92.7 | 92.9 | 93.4 |
| GEG | 6.989070 | 0.249860 | 64 | 63.6 | 63.9 | 63.8 | 63.5 |
| MAD | 6.983310 | 0.271510 | 54.8 | 55.7 | 57.8 | 56.7 | 56.7 |
| EPE | 6.962130 | 0.246050 | 43.6 | 45.4 | 46.7 | 45.9 | 44.5 |
| EET | 6.953950 | 0.245950 | 50 | 50.4 | 50.4 | 50.2 | 50.10 |
| DEM | 6.954790 | 0.261480 | 34.6 | 32.4 | 32.5 | 32.3 |  |

| Harmattan season | | | | | | | |
| --- | --- | --- | --- | --- | --- | --- | --- |
| EL | GPRS | | Number of Dwellings in exposure location | | | | |
|  | Longitude | Latitude | R1 | R2 | R3 | R4 | R5 |
| AGB | 7.098616 | 0.277741 | 73.6 | 73 | 72.9 | 73.5 | 73.4 |
| AVT | 7.088344 | 0.280118 | 54.2 | 55.6 | 53.2 | 54.2 | 55.3 |
| TKR | 7.088187 | 0.272883 | 64.5 | 65.3 | 65.1 | 64.9 |  |
| DGE | 7.091226 | 0.263588 | 90.8 | 90.9 | 90.3 | 91.4 |  |
| KPO | 7.084466 | 0.255666 | 83.4 | 82.9 | 82.6 | 83 |  |
| KDA | 7.067490 | 0.261050 | 67.8 | 68 | 68.4 | 67.9 | 67.5 |
| KOF | 7.067070 | 0.276260 | 129 | 128.5 | 129.4 | 129.1 |  |
| TTD | 7.061200 | 0.289890 | 64.8 | 65.7 | 66.2 | 65.9 |  |
| GBB | 7.057950 | 0.250910 | 87.7 | 88.9 | 88.9 | 88.9 |  |
| AAG | 7.060880 | 0.237290 | 92 | 91.8 | 91.7 | 92.3 |  |
| DDG | 7.049880 | 0.238450 | 57 | 56.7 | 56 | 56.9 |  |
| KTD | 7.052080 | 0.272250 | 34 | 33.5 | 34.4 | 34.1 | 33.9 |
| VVA | 7.044420 | 0.270770 | 54.5 | 55 | 55 | 54.9 | 55.2 |
| RRK | 7.029540 | 0.242780 | 40.8 | 40.6 | 40.9 | 40.1 | 40.1 |
| PPO | 7.018850 | 0.249220 | 50.3 | 50.9 | 50.7 | 50.9 | 50.9 |
| FFK | 7.004800 | 0.271830 | 72.7 | 73 | 73.3 | 73.1 | 73.2 |
| GGO | 7.025450 | 0.222080 | 32.3 | 32 | 32.3 | 33.2 | 33 |
| KST | 7.015280 | 0.223030 | 151.4 | 150.7 | 150.1 | 149.9 | 151.20 |
| SSK | 7.003960 | 0.230740 | 99.2 | 99.4 | 99.1 | 98.9 | 99 |
| FSK | 7.009200 | 0.245840 | 96.1 | 97.1 | 96.3 | 97.3 |  |
| ABK | 7.001650 | 0.246370 | 87.6 | 88 | 88.3 | 87.5 | 88.10 |
| GEG | 6.989070 | 0.249860 | 55 | 55.1 | 54.5 | 55.4 | 54.9 |
| MAD | 6.983310 | 0.271510 | 46.7 | 47 | 46.9 | 46.6 | 46.8 |
| EPE | 6.962130 | 0.246050 | 36.5 | 36.7 | 34.8 | 35.8 | 35 |
| EET | 6.953950 | 0.245950 | 47.8 | 48 | 47.9 | 47.7 | 47.50 |
| DEM | 6.954790 | 0.261480 | 31.1 | 30.8 | 32 | 31.6 |  |

| Annual | | | | | | | |
| --- | --- | --- | --- | --- | --- | --- | --- |
| EL | GPRS | | Number of Dwellings in exposure location | | | | |
|  | Longitude | Latitude | R1 | R2 | R3 | R4 | R5 |
| AGB | 7.098616 | 0.277741 | 76.7 | 75.3 | 76.1 | 75.7 | 75.9 |
| AVT | 7.088344 | 0.280118 | 56.8 | 56.1 | 57.0 | 56.9 | 57.4 |
| TKR | 7.088187 | 0.272883 | 88.0 | 87.9 | 87.5 | 88.4 |  |
| DGE | 7.091226 | 0.263588 | 96.7 | 96.4 | 95.9 | 94.7 |  |
| KPO | 7.084466 | 0.255666 | 78.5 | 77.8 | 77.2 | 78.3 |  |
| KDA | 7.067490 | 0.261050 | 111.9 | 112.5 | 113.2 | 113.5 | 111.7 |
| KOF | 7.067070 | 0.276260 | 101.5 | 101.7 | 101.0 | 101.3 |  |
| TTD | 7.061200 | 0.289890 | 67.8 | 66.9 | 67.2 | 67.0 |  |
| GBB | 7.057950 | 0.250910 | 91.4 | 91.8 | 91.1 | 90.1 |  |
| AAG | 7.060880 | 0.237290 | 86.4 | 86.4 | 85.6 | 85.2 |  |
| DDG | 7.049880 | 0.238450 | 58.3 | 58.0 | 59.4 | 59.5 |  |
| KTD | 7.052080 | 0.272250 | 25.2 | 25.9 | 26.1 | 26.4 | 25.8 |
| VVA | 7.044420 | 0.270770 | 69.7 | 69.3 | 69.0 | 69.5 | 70.0 |
| RRK | 7.029540 | 0.242780 | 44.8 | 45.0 | 44.2 | 44.3 | 45 |
| PPO | 7.018850 | 0.249220 | 47.1 | 48.0 | 48.7 | 48.0 | 47.0 |
| FFK | 7.004800 | 0.271830 | 62.3 | 62.6 | 63.0 | 63.4 | 63.9 |
| GGO | 7.025450 | 0.222080 | 37.1 | 37.8 | 37.2 | 38.2 | 38 |
| KST | 7.015280 | 0.223030 | 144.4 | 143.9 | 144 | 145.2 | 144.2 |
| SSK | 7.003960 | 0.230740 | 103.7 | 102.2 | 103.1 | 103.8 | 103.0 |
| FSK | 7.009200 | 0.245840 | 89.0 | 88.9 | 89.3 | 88.5 |  |
| ABK | 7.001650 | 0.246370 | 42.4 | 42.6 | 43.3 | 43.3 | 43.0 |
| GEG | 6.989070 | 0.249860 | 54.3 | 54.9 | 54.6 | 55.7 | 56.1 |
| MAD | 6.983310 | 0.271510 | 87.1 | 85.1 | 85.3 | 85.6 | 86 |
| EPE | 6.962130 | 0.246050 | 35.8 | 35.2 | 35.4 | 34.1 | 36 |
| EET | 6.953950 | 0.245950 | 38.0 | 38.3 | 38.2 | 38.0 | 38.2 |
| DEM | 6.954790 | 0.261480 | 29.0 | 29.4 | 29.0 | 28.8 |  |
